# Metabolic modelling of polyhydroxyalkanoate copolymers production by mixed microbial cultures

**DOI:** 10.1186/1752-0509-2-59

**Published:** 2008-07-08

**Authors:** João ML Dias, Adrian Oehmen, Luísa S Serafim, Paulo C Lemos, Maria AM Reis, Rui Oliveira

**Affiliations:** 1 REQUIMTE, Chemistry Department, FCT/Universidade Nova de Lisboa, 2829-516 Caparica, Portugal

## Abstract

**Background:**

This paper presents a metabolic model describing the production of polyhydroxyalkanoate (PHA) copolymers in mixed microbial cultures, using mixtures of acetic and propionic acid as carbon source material. Material and energetic balances were established on the basis of previously elucidated metabolic pathways. Equations were derived for the theoretical yields for cell growth and PHA production on mixtures of acetic and propionic acid as functions of the oxidative phosphorylation efficiency, P/O ratio. The oxidative phosphorylation efficiency was estimated from rate measurements, which in turn allowed the estimation of the theoretical yield coefficients.

**Results:**

The model was validated with experimental data collected in a sequencing batch reactor (SBR) operated under varying feeding conditions: feeding of acetic and propionic acid separately (control experiments), and the feeding of acetic and propionic acid simultaneously. Two different feast and famine culture enrichment strategies were studied: (i) either with acetate or (ii) with propionate as carbon source material. Metabolic flux analysis (MFA) was performed for the different feeding conditions and culture enrichment strategies. Flux balance analysis (FBA) was used to calculate optimal feeding scenarios for high quality PHA polymers production, where it was found that a suitable polymer would be obtained when acetate is fed in excess and the feeding rate of propionate is limited to ~0.17 C-mol/(C-mol.h). The results were compared with published pure culture metabolic studies.

**Conclusion:**

Acetate was more conducive toward the enrichment of a microbial culture with higher PHA storage fluxes and yields as compared to propionate. The P/O ratio was not only influenced by the selected microbial culture, but also by the carbon substrate fed to each culture, where higher P/O ratio values were consistently observed for acetate than propionate. MFA studies suggest that when mixtures of acetate and propionate are fed to the cultures, the catabolic activity is primarily guaranteed through acetate uptake, and the characteristic P/O ratio of acetate prevails over that of propionate. This study suggests that the PHA production process by mixed microbial cultures has the potential to be comparable or even more favourable than pure cultures.

## Background

Polyhydroxyalkanoates are biopolymers with physico-chemical properties similar to polypropylene but with the advantage of being biodegradable and biocompatible. Industrial PHA production technology is currently based on bacteria cultivation using pure cultures grown in well-defined nutrient deficient synthetic media with single substrates [1]. The relatively high production costs constitute presently the most important barrier to PHA becoming a commodity polymer in direct competition with oil-based polymers. The cost of the substrate and of the equipment required for aseptic operation is responsible for about 40% of the total PHA production cost [1].

Potential strategies for reducing both the operational and capital expenses of PHA production are the use of open mixed cultures of microorganisms instead of pure cultures, and the use of waste materials as the carbon substrate. Activated sludge is known to accumulate up to 65% PHA per cell dry weight [2-4]. There are currently many possibilities of industrial and agricultural wastes that could be directed to the production of PHA on the basis of open mixed microbial systems [5,6].

**Figure 1:**
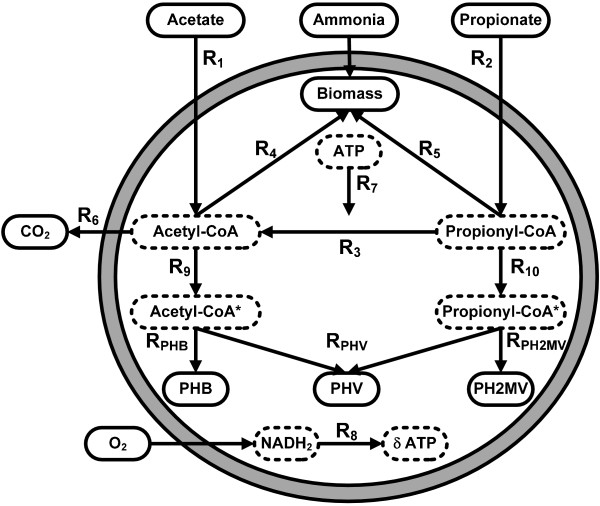
**PHA metabolic network**. Figure shows schematic representation of the metabolic reactions, substrates, products (both marked by full lines) and metabolites (marked by dashed lines) involved in PHA production by mixed microbial cultures.

Mixed microbial cultures cannot directly metabolize sugars into PHA. A pre-fermentation step is required in order to convert sugars into volatile fatty acids (VFA) such as acetic, propionic, valeric and butyric acid. This pre-fermentation step can, however, be accomplished with high yield [5,7]. The final PHA molecular structure is highly dependent on the VFA composition used as carbon source. The most well-studied case is the production of polyhydroxybutyrate (PHB), a homopolymer, obtained when acetic acid is used as the sole carbon source. The metabolism for this transformation (included in Figure 1) can be defined by 6 basic reactions [8,9]: acetate uptake (R_1_), growth on acetyl-CoA (R_4_), catabolism (R_6_), oxidative phosphorylation (R_8_), maintenance (R_7_) and PHB production (R_PHB_). A metabolic model based on these reactions was comprehensively validated with experimental data [10]. This study demonstrated an excellent agreement between the model predictions and experimental data.

The properties and quality of PHA are highly dependent on the monomeric composition, which, in turn, is dependent on the VFA mixture adopted as carbon source. PHB is a relatively low quality polymer since it is too brittle for many applications [1,11]. In terms of PHA processing, the production of copolymers appears to be of higher commercial interest [12-14]. The production of PHB homopolymer and copolymers of 3-hydroxybutyrate (3HB), 3-hydroxyvalerate (3HV) and 3-hydroxy-2-methylvalerate (3H2MV) from mixtures of acetic and propionic acid has been studied by several researchers [11,15-17]. There are, however, no metabolic models currently available describing the production of such copolymers. Metabolic models can be used to facilitate process optimization, and also serve as a reference basis in the interpretation of data arising from the study of biological processes. The comparison of experimentally-determined stoichiometry with the theoretical model predictions allows a better understanding of the processes under study. The main objective of this work was to extend existing PHB models [8,10] to the production of PHA copolymers from mixtures of VFA. This study will be focused on the PHA production phase, and is confined to mixtures of acetic and propionic acid, which are seen as the most promising for the synthesis of high quality PHAs [11,18].

### Metabolic model

A schematic of the metabolic model developed in this study is shown in Figure 1. Acetate and propionate are taken up inside the cell, and used for PHA production and biomass growth. Energy is generated within the cells through oxidative phosphorylation, while a portion of this energy is also necessary for cell maintenance. A detailed description of the biochemical reactions, material and energy balances, kinetic reactions and metabolic fluxes is provided below.

### Basic reactions

The detailed biochemical reactions involved in the PHA production process using acetate and propionate carbon sources under aerobic conditions are shown in Table 1. All reactions are expressed on a carbon-mole basis. In the feast phase, acetate and propionate are taken up inside the cells by means of active transport, requiring one mole of ATP per mole of carbon source. They are then converted into acetyl-CoA and propionyl-CoA, respectively, at the cost of another mole of ATP per mole of acetate or propionate. The net reactions are shown in R_1 _and R_2 _[19].

**Table 1:** Metabolic model of PHA production from mixtures of acetate and propionate by mixed microbial cultures

**Reaction**	**Stoichiometry**
**R_1_: Acetate Uptake**	CH_2_O + ATP → CHO_0.5 _+ 0.5·H_2_O
**R_2_: Propionate Uptake**	CH2O23 + 0.67·ATP → CH43O13 + 0.33·H_2_O
**R_3_: Propionyl-CoA converted to Acetyl-CoA**	1.5·CH43O13 + H_2_O → CHO_0.5 _+ 1.5·NADH_2 _+ 0.5·CO_2_
**R_4_: Growth on Acetyl-CoA**	1.27·CHO_0.5 _+ 0.2·NH_3 _+ K_1_·ATP + 0.3·H_2_O → CH_1.4_N_0.2_O_0.4 _+ 0.53·NADH_2 _+ 0.27·CO_2_
**R_5_: Growth on Propionyl-CoA**	1.06·CH43O13 + 0.2·NH_3 _+ K_2_·ATP + 0.17·H_2_O → CH_1.4_N_0.2_O_0.4 _+ 0.47·NADH_2 _+ 0.06·CO_2_
**R_6_: Catabolism**	CHO_0.5 _+ 1.5·H_2_O → CO_2 _+ 2·NADH_2 _+ 0.5·ATP
**R_7_: Maintenance**	ATP → m_ATP_
**R_8_: Oxidative Phosphorylation**	NADH_2 _+ 0.5·O_2 _→ H_2_O+δ·ATP
R_9_: Acetyl-CoA* Production	CHO_0.5 _+ 0.25·NADH_2 _→ CH_1.5_O_0.5_
R_10_: Propionyl-CoA* Production	CH43O13 + 0.17·NADH_2 _→ CH53O13
**R_PHB_: PHB Production**	CH_1.5_O_0.5 _→ PHB
**R_PHV_: PHV Production**	0.4·CH_1.5_O_0.5 _+ 0.6·CH53O13 → PHV
**R_PH2MV_: PH2MV Production**	CH53O13 → PH2MV

While acetate is converted to acetyl-CoA and propionyl-CoA is produced from propionate, it has also been observed that a portion of the propionyl-CoA produced from propionate uptake is converted into acetyl-CoA [11]. This transformation may potentially proceed through 5 different biochemical pathways [11], however, the net material and reducing power transformations are identical through each pathway. The difference between the pathways lies only in the amount of energy generated. For the purposes of this model, it was assumed that propionyl-CoA was first converted to succinyl-CoA via the methylmalonyl-CoA pathway, then converted to oxaloacetate, pyruvate and acetyl-CoA. The net reaction is described in R_3_.

In PHA production, acetyl-CoA and propionyl-CoA are reduced and condensed to produce a polymer consisting of various 3-hydroxyalkanoates (3HAs) monomers. 3HB is formed from 2 acetyl-CoA monomers, one acetyl-CoA and one propionyl-CoA combine to form either 3HV or 3-hydroxy-2-methylbutyrate (3H2MB) (which are isomers of each other, and henceforth expressed only as 3HV, indicating the sum of these 2 compounds), while 2 propionyl-CoA monomers form 3H2MV. The fraction of each PHA monomer produced by the cells depends on the fluxes of acetyl-CoA and propionyl-CoA available for polymer production. It also depends on whether the cells tend to reduce and condense acetyl-CoA and propionyl-CoA in a random fashion, or whether preferential binding occurs between e.g. acetyl-CoA and propionyl-CoA. For the purposes of metabolic model development, however, it is necessary to express each reaction in relation to the overall mass, energy and redox transformations. Thus, it is more convenient to express the various potential PHA fractions produced by the cells in terms of the reduced and condensed monomers produced from acetyl-CoA or propionyl-CoA. As shown in R_9 _and R_10_, these monomers are represented as acetyl-CoA* and propionyl-CoA*, respectively, in a similar fashion as expressed by a previous study [20]. The PHA formation process from acetyl-CoA* and propionyl-CoA* has no further demand for energy or reducing power.

Biomass growth occurs from acetyl-CoA and propionyl-CoA, respectively. The energy required for the production of 1 C-mol of biomass from acetyl-CoA is represented as K_1_, which has been estimated as 1.7 mol ATP [21]. The reaction of biomass growth from acetyl-CoA was determined through stoichiometric and redox balancing, and is shown in R_4_. The amount of energy required for biomass synthesis from propionyl-CoA is represented as K_2_, which has been estimated to be 1.38 mol ATP per C-mol of biomass formed [22]. Similarly, it was assumed that propionyl-CoA is converted to succinyl-CoA for biomass synthesis [22]. The net reaction is shown in R_5_. The biomass formula was assumed to be CH_1.4_O_0.4_N_0.2 _[23].

Catabolism of acetyl-CoA and propionyl-CoA also occurs in the microbial cells. Acetyl-CoA is converted to CO_2 _through the tricarboxylic acid cycle (TCA) as described in R_6 _[19]. The catabolism of propionyl-CoA is assumed to occur via acetyl-CoA, where R_3 _is followed by R_6_.

Energy in the form of ATP is produced from NADH_2 _through oxidative phosphorylation. The amount of ATP generated per mole of NADH_2 _oxidized is expressed by the P/O ratio, δ, which represents the efficiency of oxidative phosphorylation [24]. This reaction is expressed in R_8_.

In addition to the energetic transformations described above, energy is also required for cell maintenance. The rate of ATP consumption for maintenance purposes is described in R_7 _as m_ATP_.

### Pseudo steady-state material balancing of intracellular intermediates

The metabolic network of Figure 1 has *q *= 13 metabolic reactions, *m *= 6 intracellular metabolites (marked by dashed lines), 4 input substrates (acetic and propionic acid, ammonia and oxygen) and 5 end-products [biomass, PHB, poly(3-hydroxyvalerate) (PHV), poly(2-methyl-3-hydroxyvalerate) (PH2MV) and CO_2_]. The steady state material balances to the *m *intracellular intermediates (acetyl-CoA, acetyl-CoA*, propionyl-CoA, propionyl-CoA*, NADH_2 _and ATP) are:

(1a) **Ac-CoA**: *R*_1 _+ *R*_3 _- 1.27·*R*_4 _- *R*_6 _- *R*_9_= 0

(1b) Ac-CoA*: *R*_9 _- *R*_*PHB *_- 0.4·*R*_*PHV *_= 0

(1c) **Prop-CoA**: *R*_2 _- 1.5·*R*_3 _- 1.06·*R*_5 _- *R*_10 _= 0

(1d) Prop-CoA*: *R*_10 _- 0.6·*R*_*PHV *_- *R*_*PH*2*MV *_= 0

(1e) **NADH_2_**: 1.5·*R*_3 _+ 0.53·*R*_4 _+ 0.47·*R*_5 _+ 2·*R*_6 _- *R*_8 _- 0.25·*R*_9 _- 0.17·*R*_10 _= 0

(f) **ATP**: -*R*_1 _- 0.67·*R*_2 _- *K*_1_·*R*_4 _- *K*_2_·*R*_5 _+ 0.5·*R*_6 _- *R*_7 _+ δ·*R*_8 _= 0

### Constraints to the metabolic network

From acetyl-CoA* and propionyl-CoA*, the resulting PHA polymer can be comprised of either PHB (2 acetyl-CoA* molecules), PHV (one acetyl-CoA* and one propionyl-CoA*) and PH2MV (2 propionyl-CoA*). The PHA composition depends on whether selective or random condensation of acetyl-CoA* and propionyl-CoA* takes place. In the propionate enriched culture selective condensation was observed, where acetyl-CoA* preferentially bounds with propionyl-CoA*, forming PHV [11]. Since acetyl-CoA* was generated in higher abundance than propionyl-CoA*, the remaining acetyl-CoA* condensed to form PHB, while PH2MV was not produced by the sludge. This selective condensation is expressed by the following three additional equations:

(2a) $$R_{PHB}=R_{9}-\frac{2}{3}\cdot R_{10}$$

(2b) $$R_{PHV}=\frac{5}{3}\cdot R_{10}$$

(2c) *R*_*PH*2*MV *_= 0

since *R*_*PH*2*MV *_= 0 (Eq. 2c), Eqs. (2a–b) are linearly dependent to Eqs. (1a–f), only a single additional constraint is introduced with R_PH2MV _= 0.

In the acetate enriched culture, the PHA composition consisted of a lower PHV fraction and a higher PH2MV and PHB fraction, as is expected by microorganisms performing random condensation of acetyl-CoA* and propionyl-CoA*. In this latter case the following three equations are applied [20]:

(3a) $$R_{PHB}=\frac{2\cdot R_{9}^{2}}{2\cdot R_{9}+3\cdot R_{10}}$$

(3b) $$R_{PHV}=\frac{5\cdot R_{9}\cdot R_{10}}{2\cdot R_{9}+3\cdot R_{10}}$$

(3c) $$R_{PH2MV}=\frac{3\cdot R_{10}^{2}}{2\cdot R_{9}+3\cdot R_{10}}$$

The *R*_9 _and *R*_10 _fluxes are sufficient to calculate the fluxes of *R*_*PHB*_, *R*_*PHV *_and *R*_*PH*2*MV*_.

On the other hand, constraints (3a–c) automatically obey the material balances (1a–f) (see [20] for details), thus also a single additional constraint can also be added to the material balances here (1a–f).

### Dynamic material balancing of substrates and end-products

The transient material balances in a batch system of input substrates (acetate, propionate, active biomass, ammonia) and intracellular contents of PHB, PHV and PH2MV are the following:

(4a) $$\frac{dAc}{dt}=-R_{1}\cdot X$$

(4b) $$\frac{d\mathrm{Pr}op}{dt}=-R_{2}\cdot X$$

(4c) $$\frac{dN}{dt}=-0.2\cdot (R_{4}+R_{5})\cdot X$$

(4d) $$\frac{dX}{dt}=(R_{4}+R_{5})\cdot X$$

(4e) $$\frac{df_{PHB}}{dt}=R_{PHB}-(R_{4}+R_{5})\cdot f_{PHB}$$

(4f) $$\frac{df_{PHV}}{dt}=R_{PHV}-(R_{4}+R_{5})\cdot f_{PHV}$$

(4g) $$\frac{df_{PH2MV}}{dt}=R_{PH2MV}-(R_{4}+R_{5})\cdot f_{PH2MV}$$

### Theoretical yields

The theoretical yields and VFA and oxygen maintenance coefficients were derived analytically from the material balance equations (1a–f). The expressions are compiled in Table 2. The theoretical yield coefficients are functions of δ, *K*_1_, *K*_2 _and *y*, the ratio of propionate flux to total carbon flux (see Eq. 5):

**Table 2:** Theoretical yields and maintenance coefficients

**Process description**	**VFA**	**Oxygen**
**Cell growth on acetate**	YX,AcS=(4⋅δ+0.67⋅y⋅δ+0.33⋅y−1)(4⋅δ+2⋅K1+1.27)	YX,AcO2=(4⋅δ+0.67⋅y⋅δ+0.33⋅y−1)(0.33⋅y⋅K1−0.12⋅y+2.27+2⋅K1)
**Cell growth on propionate**	YX,Pr⁡opS=(4⋅δ+0.67⋅y⋅δ+0.33⋅y−1)(4⋅δ+2⋅K2+0.71)	YX,Pr⁡opO2=(4⋅δ+0.67⋅y⋅δ+0.33⋅y−1)(0.33⋅y⋅K2−0.22⋅y+1.71+2⋅K2)
**PHB Storage**	YPHBS=(4⋅δ+0.67⋅y⋅δ+0.33⋅y−1)(4⋅5⋅δ+1)	YPHBO2=(4⋅δ+0.67⋅y⋅δ+0.33⋅y−1)(2.12−0.21⋅y)
**PHV Storage**	YPHVS=(4⋅δ+0.67⋅y⋅δ+0.33⋅y−1)(4⋅8⋅δ+0.8)	YPHVO2=(4⋅δ+0.67⋅y⋅δ+0.33⋅y−1)(2−0.27⋅y)
**PH2MV Storage**	YPH2MVS=(4⋅δ+0.67⋅y⋅δ+0.33⋅y−1)(5⋅δ+0.67)	YPH2MVO2=(4⋅δ+0.67⋅y⋅δ+0.33⋅y−1)(1.92−0.31⋅y)
**Maintenance**	$m_{S}=\frac{2}{\left(4\cdot \delta +0.67\cdot y\cdot \delta +0.33\cdot \text{y}-1\right)}\cdot m_{ATP}$	$m_{O2}=\frac{\left(2+0.33\cdot y\right)}{\left(4\cdot \delta +0.67\cdot y\cdot \delta +0.33\cdot \text{y}-1\right)}\cdot m_{ATP}$

(5) $$y=\frac{R_{2}}{R_{1}+R_{2}}$$

### Kinetic model

As stated above, the metabolic network has *q *= 13 fluxes and a total number of 7 constraints. Therefore, at least 6 fluxes must be defined *a priori *in order to calculate the network fluxes. Table 3 compiles the kinetic equations used in this work to model the missing fluxes. This model is an extension of a previous acetate to PHB metabolic model [10]. Briefly, the model defines the following kinetic rates:

**Table 3:** Kinetic model

**Reaction**	**Kinetics**
**R_4_: Growth on Acetyl-CoA**	$R_{4}=R_{4,\max}\cdot \frac{Ac}{Ac+K_{S}}\cdot \frac{N}{N+K_{N}}$
**R_5_: Growth on Propionyl-CoA**	$R_{5}=R_{5,\max}\cdot \frac{\mathrm{Pr}op}{\mathrm{Pr}op+K_{S}}\cdot \frac{N}{N+K_{N}}$
**R_6_: Catabolism**	$R_{6}=R_{6,\max}\cdot \frac{S}{S+K_{S}}$
**R_7_: Maintenance**	$R_{7}=R_{7,\max}\cdot \frac{S}{S+K_{S}}$
R_9_: Acetyl-CoA* Production	$R_{9}=\max\left\{R_{9,\max}\cdot \frac{S}{S+K_{S}}\cdot \left[1-{\left(\frac{f_{PHA}}{f_{PHA,\max}}\right)}^{\alpha }\right],\frac{2}{3}\cdot R_{10}\right\}$
R_10_: Propionyl-CoA* Production	$R_{10}=R_{10,\max}\cdot \frac{S}{S+K_{S}}\cdot \left[1-{\left(\frac{f_{PHA}}{f_{PHA,\max}}\right)}^{\alpha }\right]$

• Growth on acetyl-CoA (*R*_4_) is limited by the concentrations of acetate and ammonia.

• Growth on propionyl-CoA (*R*_5_) is limited by the concentrations of propionate and ammonia.

• Catabolism (*R*_6_) is limited by total VFA concentration. The kinetic equation for catabolism is only used for mixtures of acetate and propionate to fulfil the 6 fluxes required to perform MFA.

• Maintenance (*R*_7_) is limited by total VFA concentration.

• Acetyl-CoA* synthesis (*R*_9_) is limited by total VFA concentration and inhibited by the intracellular PHA content. Intracellular PHA inhibition has been validated experimentally by several authors [8-10,25-27].

• Propionyl-CoA* synthesis (*R*_10_) is limited by propionate concentration and inhibited by the intracellular PHA content.

## Methods

### Reactors operation

In this study, two SBR for the production of PHA, were operated over four years. The sludge in each reactor was adapted to either acetate or propionate as the sole carbon source. The reactors working volume was 1 litre and the total SBR cycle duration was 12 h, consisting of 10.5 h of aerobiosis, 1 h of settling (agitation and air sparging switched off) and 0.5 h to withdraw half of the volume, which was replaced by the same volume of fresh medium during the first 15 min at the beginning of the next cycle. The hydraulic retention time (HRT) was therefore 1 day. At the end of each cycle, before settling, a defined volume of biomass was removed to keep the sludge retention time (SRT) at 10 days. Oxygen was supplied by an air compressor through a ceramic membrane disperser introduced inside the reactor at an airflow rate of 1.0 vvm (volume air/(volume reactor.min)), allowing for the dissolved oxygen (DO) concentration to be around 80% of the saturation value. The reactors were operated without pH control but its value was monitored on-line and ranged between 8.0 and 9.2; the temperature was controlled at 22°C and the stirring rate at 250 rpm.

### Culture medium

The standard medium used in the SBRs was composed of (per litre of distilled water): 1.269 g CH_3_CH_2_COOH or 4.0796 g of CH_3_COONa.3H_2_O, 600 mg MgSO_4_.7H_2_O, 160 mg NH_4_Cl, 100 mg EDTA, 92 mg K_2_HPO_4_, 45 mg KH_2_PO_4_, 70 mg CaCl_2_.2H_2_O and 2 ml of trace elements solution. The trace solution consisted of (per litre of distilled water): 1500 mg FeCl_3_.6H_2_O, 150 mg H_3_BO_3_, 150 mg CoCl_2_.6H_2_O, 120 mg MnCl_2_.4H_2_O, 120 mg ZnSO_4_.7H_2_O, 60 mg Na_2_MoO_4_.2H_2_O, 30 mg CuSO_4_.5H_2_O and 30 mg of KI. Thiourea (10 mg/l) was added to inhibit nitrification. The pH of the salt solution was adjusted to 7.2 and then sterilized, where the phosphorus components of the solution was sterilized separately. After sterilization, the two solutions were allowed to cool, and were then mixed together.

In the batch experiments, different concentrations of acetate, propionate and ammonia were tested for the two systems and different feeding regimens were used. For the acetate reactor: the acetate concentrations tested with 1.4 N-mmol/l of ammonia were 15 C-mmol/l, 30 C-mmol/l and 60 C-mmol/l. The ammonia concentrations with 30 Cmmol/l of acetate: 0.7 N-mmol/l, 1.4 N-mmol/l and 2.8 N-mmol/l. The feeding regimen was tested by supplying multiple pulses of 60 C-mmol/l of acetate (3 tests with 3 pulses and 1 test with 4 pulses), where 0.7 N-mmol/l of ammonia was also added in the first pulse for these tests. Two more assays were performed by supplying a 30 C-mmol/l of propionate and a mixture of acetate and propionate with 15 C-mmol/l each. The batch tests performed in the propionate system analyzed the effect of different concentrations of propionate (30 C-mmol/l, 60 C-mmol/l, 90 C-mmol/l and 120 C-mmol/l) with 1.4 N-mmol/l of ammonia and the use of acetate (in one pulse of 30 C-mmol/l and mixed with propionate, 15 C-mmol/l each). In total, 18 batch tests were performed with the two systems.

### Analytical techniques

Cell dry weight was determined as volatile suspended solids (VSS), according to Standard Methods [28]. Acetate and propionate were analyzed by HPLC using a BioRad Aminex HPX-87H column, with 0.01 N sulphuric acid as eluent, an elution rate of 0.6 ml/min and an operating temperature of 50°C. A UV detector (Merck) set at 210 nm was used. Prior to injection, samples were filtered using a 0.2 μm membrane. PHA were determined by GC after acidic estherification (see [4,11] for details). Ammonia was analyzed using an ammonia gas sensing combination electrode (ThermoOrion 9512).

### Parameter estimation

The kinetic parameters were estimated by non-linear weighted least-squares (MATLAB's *lsqnonlin *function) using the Levenberg-Marquardt algorithm. The program minimized the root mean squared error:

(6) $$rmse=\sqrt{\frac{e^{T}\cdot e}{\left(n-p\right)}}$$

with **e **the vector of residuals scaled by their maximum values, *n *the number of measurements and *p *the number of parameters to estimate.

The confidence bounds of the parameters were estimated by the approximation of the Hessian matrix, **H**, by the Jacobian matrix, **J**, at the minimum root mean squared error, *rmse*.

(7) **H **= **J**^**T**^·**J**·*rmse*

Finally, confidence bounds, CB_p_, were obtained by the estimate of standard deviations for a level of confidence of 95%:

(8) **CB **= diag(**H**)·t(1 - 0.95, *n *- *p*)

with *t *the t-student distribution.

For the calculation of residuals, Eqs. (4a–g) were integrated using a 4^th^/5^th ^order Runge-Kutta solver (MATLAB's *ode45 *function), with the obtained concentrations of acetate, propionate, PHB, PHV, PH2MV and oxygen subtracted from the respective measured values to give the residuals.

### Metabolic Flux Analysis

Metabolic Flux Analysis (MFA) is a methodology that allows the determination of a set of unknown metabolic fluxes, **v**_**n**_, from a set of known fluxes, **v**_**b **_[29] by steady-state material balancing (here using Eqs. (1a–f)). In the present case, **v**_**b **_is composed of the modelled fluxes:

**v**_**b **_= [*R*_4_, *R*_5_, *R*_6_, *R*_7_, *R*_9_, *R*_10_]^T^

The remaining fluxes were calculated by solving the central MFA equation [29]:

**v**_**n **_= -**A**_**n**_^-1^·**A**_**b**_·**v**_**b**_

with **v**_**n **_the vector of unknown fluxes,

**v**_**n **_= [*R*_1_, *R*_2_, *R*_3_, *R*_8_, *R*_*PHB*_, *R*_*PHV*_, *R*_*PH*2*MV*_]^T^

**A**_**n **_and **A**_**b **_are the corresponding stoichiometric matrices of the unknown and known fluxes respectively. Note that in this case **A**_**n **_is a square (7 × 7) matrix.

For single substrate feeding studies, either R_1 _or R_2 _equals zero, thus only 5 modelled

fluxes had to be included in **v**_**b**_, being R_6 _moved to **v**_**n**_.

### Flux Balance Analysis

Flux Balance Analysis (FBA) is a method to optimize a given metabolic objective function under the constraints of steady-state material balancing of intracellular intermediates and other known biological constraints [30]. FBA was applied in this study to maximize the flux of PHA synthesis with a desired 24% (C-mol/C-mol) propionyl-CoA* composition. Mathematically, the problem can be stated in the following way.

(12a) $$\underset{R_{1},R_{2}}{\max}\left(R_{PHB}+R_{PHV}+R_{PH2MV}\right)$$

under the following constraints:

(12b) **0 **= **A**_**n**_·**v**_**n **_+ **A**_**b**_·**v**_**b**_

(12c) 0 = *R*_*PHB *_+*R*_*PHV *_+ *R*_*PH*2*MV *_- 0.76·*R*_9 _- 0.24·*R*_10_

(12d) *R*_4 _= *R*_5 _= 0

(12e) *R*_7 _= 0.02

(12f) $$\frac{R_{3}}{R_{2}}=0.43\frac{R_{2}}{R_{1}+R_{2}}$$

Constraint (12c) defines the desired monomeric composition of the final polymer. Constraint (12d) states that the cells are not growing during the PHA synthesis phase. The maintenance flux (Eq. 12e) was set to a known flux (see Table 4). Constraint (12f) was established on the basis of experimental observations and discussed later in the results section. FBA problems are normally solved through linear programming using the simplex algorithm. In our case, constraint (12f) is a nonlinear equation, thus a nonlinear function solver was adopted. The MATLAB function '*fmincon*' based on the quasi-Newton optimization algorithm was adopted to solve the system (12a–f).

**Table 4:** Parameters estimation results of acetate and propionate enriched cultures for the different feeding conditions

**Substrate**	**Acetate**		**Propionate**		**Mixture**	
**Culture enrichment**	**Acetate**	**Propionate**	**Acetate**	**Propionate**	**Acetate**	**Propionate**

**Number of experiments used for parameters estimation/model validation**	(7/3)	(1/0)	(1/0)	(3/1)	(1/0)	(1/0)

**P/O ratio**, d **(mol-ATP/mol-NADH**_2_**)**	2.94 ± 0.16	1.08 ± 0.12	1.80 ± 0.19	0.94 ± 0.04	2.90 ± 0.27	1.78 ± 0.19

**Maximum rates determined by metabolic model [C-mol/(C-mol.h)]**						

***R***_6, *max*_	-	-	-	-	0.092 ± 0.017	0.094 ± 0.046
***R_9, *max*_***	0.52 ± 0.03	0.13 ± 0.01	0.033 ± 0.005	0.053 ± 0.003	0.30 ± 0.03	0.18 ± 0.02
***R_10, *max*_***	-	-	0.041 ± 0.004	0.052 ± 0.003	0.22 ± 0.02	0.13 ± 0.02

**Maximum rates determined by MFA [C-mol/(C-mol.h)]**						

***R_1, *max*_***	0.73 (0.01)	0.21 (0.02)	-	-	0.33 (0.07)	0.22 (0.05)
***R_2, *max*_***	-	-	0.12 (0.02)	0.17 (0.02)	0.34 (<0.01)	0.23 (<0.01)
***R_3, *max*_***	-	-	0.049 (0.005)	0.074 (0.005)	0.074 (0.018)	0.059 (0.016)
***R_6, *max*_***	0.18 (<0.01)	0.07 (<0.01)	0.016 (<0.001)	0.021 (0.003)	-	-
***R_8, *max*_***	0.24 (0.02)	0.11 (<0.01)	0.094 (0.006)	0.13 (0.02)	0.19 (<0.01)	0.22 (0.01)
***R_*PHB*, *max*_***	0.52 (0.03)	0.13 (0.01)	0.011 (0.003)	0.018 (0.001)	0.14 (0.02)	0.090 (0.005)
***R_*PHV*, *max*_***	-	-	0.035 (0.006)	0.086 (0.006)	0.26 (0.03)	0.22 (0.03)
***R_*PH*2*MV*, *max*_***	-	-	0.026 (0.003)	-	0.12 (0.01)	-

The confidence bounds in parenthesis were obtained by error propagation of the metabolic model parameters

*f*_*PHA*, *max *_= 2.47 C-mmol/C-mmol [10]; a = 3.85 (dimensionless) [10]; *m*_*ATP *_= 0.02 mol-ATP/(C-mol.h) [39]; *K*_*S *_= 0.062 C-mmol/l [39]; *K*_*N *_= 0.56 N-mmol/l [40]

## Results and discussion

### Estimation of the kinetic parameters

The data of 18 batch experiments were used for model calibration and validation. In mixed microbial cultures there is a diversity of organisms with different phenotypes competing for the nutrients in the medium. The observed metabolic activity is thus an "average" metabolic activity over all organisms present in the culture. This metabolic activity is obviously highly dependent on the culture enrichment regimen and also on the VFA feeding strategy. As such, the parameter estimation was done separately for the following 6 distinct experimental conditions:

1. Acetate enriched culture fed with acetate.

2. Acetate enriched culture fed with propionate.

3. Acetate enriched culture fed with mixtures of acetate and propionate.

4. Propionate enriched culture fed with acetate.

5. Propionate enriched culture fed with propionate.

6. Propionate enriched culture fed with mixtures of acetate and propionate.

The estimated parameter values and respective 95% confidence limits are compiled in Table 4. Note that the estimated confidence bounds are generally quite low, denoting the high sensitivity of residuals to parameters and the high statistical confidence of the estimated parameter values.

**Figure 2:**
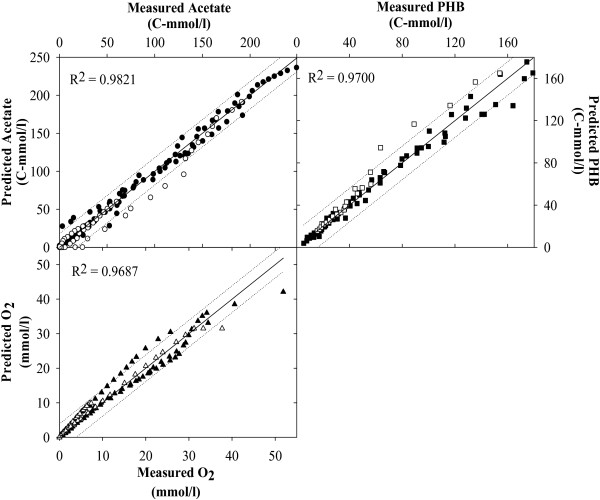
**Modelling results**. Figure shows the results obtained for acetate enriched cultures under acetate feeding conditions. Full symbols represent parameter estimation results and open symbols the model validation results. Dashed lines are the 95% confidence limits for the parameter estimation and model validation results.

**Figure 3:**
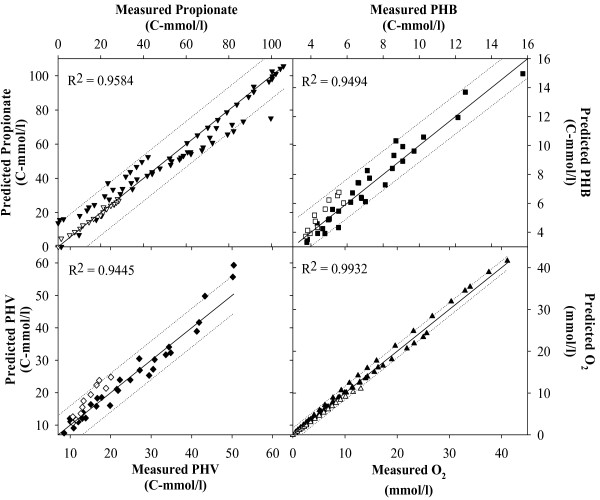
**Modelling results**. Figure shows the results obtained for propionate enriched cultures under propionate feeding conditions. Full symbols represent parameter estimation results and open symbols the model validation results. Dashed lines are the 95% confidence limits for the parameter estimation and model validation results.

**Figure 4:**
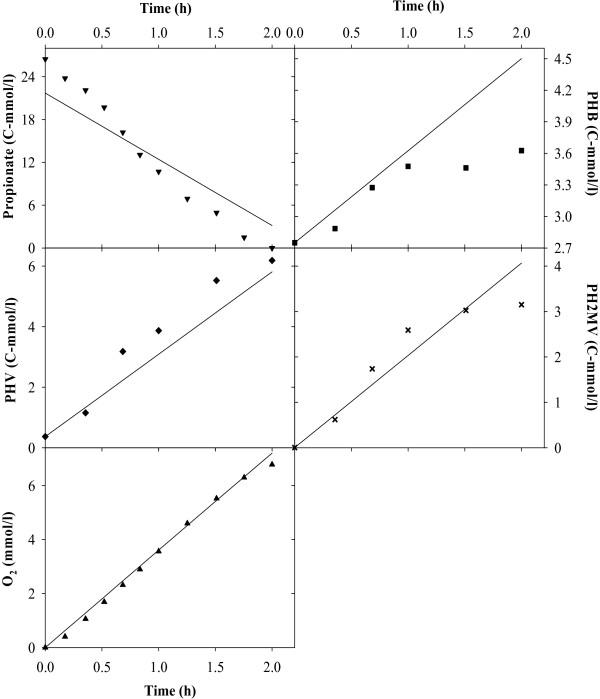
**Modelling results**. Figure shows the results obtained for acetate enriched cultures under propionate feeding conditions. Full symbols represent the experimental data and full lines the modelling results.

**Figure 5:**
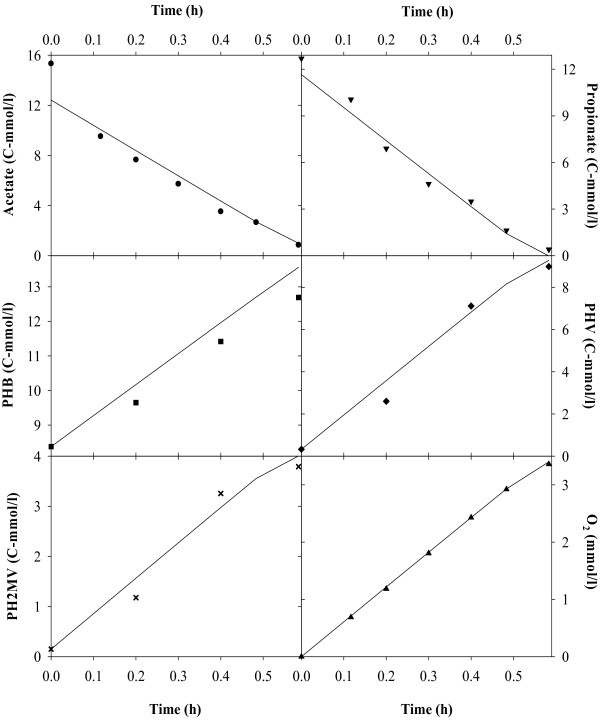
**Modelling results**. Figure shows the results obtained for acetate enriched cultures under acetate and propionate feeding conditions. Full symbols represent the experimental data and full lines the modelling results.

**Figure 6:**
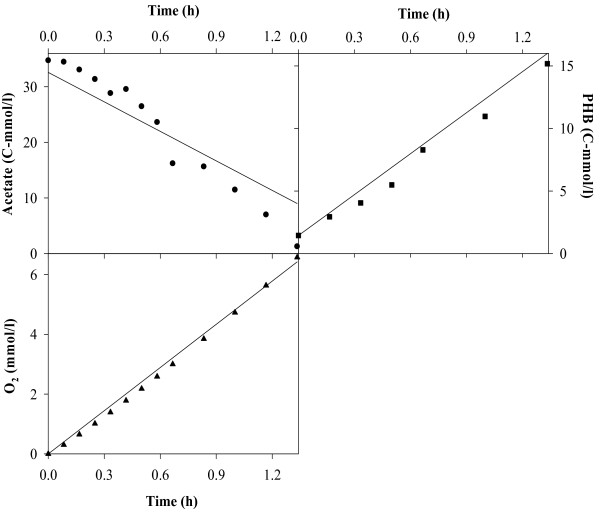
**Modelling results**. Figure shows the results obtained for propionate enriched cultures under acetate feeding conditions. Full symbols represent the experimental data and full lines the modelling results.

**Figure 7:**
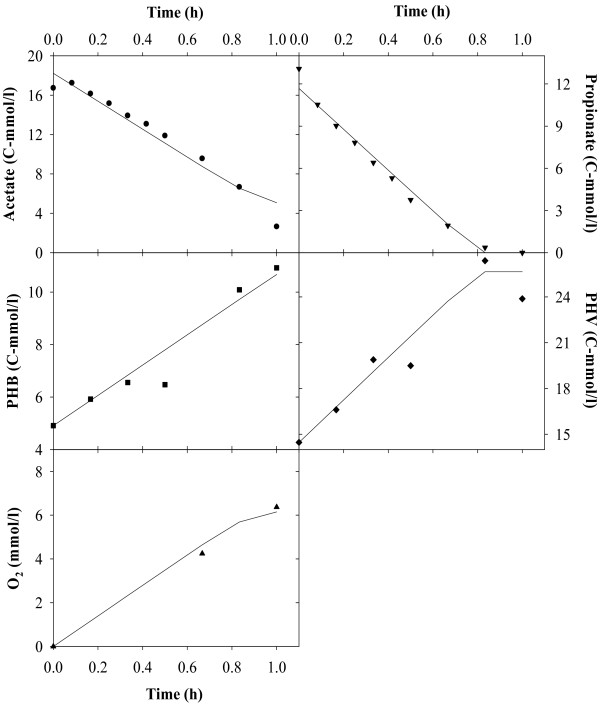
**Modelling results**. Figure shows the results obtained for propionate enriched cultures under acetate and propionate feeding conditions. Full symbols represent the experimental data and full lines the modelling results.

Figures 2, 3, 4, 5, 6, 7 compare model predictions and respective measurements for the data of the 18 experiments used in this study. The modelling results for acetate enriched cultures fed with acetate (7 calibration experiments and 3 validation experiments) and propionate enriched culture fed with propionate (3 calibration experiments and 1 validation experiment) are shown in Figures 2 and 3, respectively. Full symbols represent calibration points while open symbols denote validation points. These results show that the model was able to consistently describe the experimental data for a wide range of acetate and propionate feeding conditions, with regression coefficients, r^2^, always above 0.94. Figures 4, 5, 6, 7 show the modelling results obtained for the remaining experiments, where both cultures were fed with substrates other than those used in the enrichment phase. In these Figures, the experimental data of the acetate, propionate, PHB, PHV, PH2MV and oxygen is represented by full symbols and the model prediction by full lines. A good agreement between experimental data and model predictions was obtained in all cases.

### P/O ratio

The P/O ratio is a measure of the efficiency of ATP synthesis coupled to cell respiration, indicative of the efficiency of catabolism. In acetate enriched cultures, the P/O ratio was significantly higher than in propionate enriched cultures (Table 4). Moreover, the acetate feast and famine enrichment strategy yielded an extremely energetically effective population with a P/O ratio close to the theoretical maximum, which is between 2–3 mol-ATP/mol-NADH_2 _in bacteria [9,10,31-33]. This result may suggest that the feast and famine strategy may induce not only the accumulation of intracellular reserves but also the optimization of the global energetic efficiency of the final selected culture.

The P/O ratio results in Table 4 also show that after a short term swap in substrate, the P/O ratio is highly affected. This behaviour is common to both cultures used in the present study. When propionate is the main source of energy, the P/O ratio is consistently much lower (~1 in both acetate enriched and propionate enriched cultures) than when acetate is the main source of energy (2.9 and 1.8 in acetate and propionate enriched cultures, respectively). This result is also consistent with the generally lower energetic efficiency associated with the propionate enriched culture. Interestingly, when mixtures of acetate and propionate are fed to the culture, the characteristic P/O ratio of acetate prevails over that of propionate, which suggests that catabolic activity and respiration is preferentially executed through acetate metabolism (see discussion below for more detail).

A detailed study about the P/O ratio for microbial cells using different substrates was performed by Stouthamer [34]. In this study, a range of P/O ratios between 2.25 and 3 were found for different substrates, whereas 2.25 was the value calculated for acetate. On the other hand, Gottschalk [19] stated that P/O ratio may also vary with bacterial species and more specifically with the number of phospholylation sites they contain. In mixed cultures containing enrichments of polyphosphate and glycogen-accumulating organisms (PAO and GAO), different P/O ratios have been reported depending on the carbon source used for culture enrichment, namely acetate and propionate. The P/O ratio reported for acetate enriched cultures was 1.85 for PAO [35] and 1.73 for GAO [22], while in propionate enriched cultures the P/O ratio was reported as 1.37 for PAO and 1.29 for GAO [36]. Furthermore, it was observed that the mixed microbial culture fed with acetate exhibited a significantly lower P/O ratio (1.38) at the beginning of the reactor operational period, before the acetate culture had adapted to a feast and famine regimen (data not shown). A very high P/O ratio (2.88) was also observed for a pure culture of *Cupriavidus necator *(formerly *Ralstonia eutropha*) when imposed to the feast and famine regimen for PHA production [33]. These statements support the hypothesis that the carbon source affects the P/O ratio and the feast and famine regimen may enable the selection of bacteria with a higher P/O ratio, mainly in acetate enriched cultures.

Higher P/O ratios are normally associated with lower maintenance coefficients and higher biomass/substrate and product/substrate yields. Table 5 compiles the theoretical yields for the different culture enrichment scenarios. As expected, for the same feeding conditions a higher P/O ratio led to higher yields and lower maintenance coefficients. Within this frame, it is clear that the acetate feast and famine culture enrichment strategy produces the highest yields of cell growth and PHA formation.

**Table 5:** Yields and maintenance coefficients for acetate and propionate enriched cultures for the different feeding conditions

**Cultures enrichment**	**Acetate**			**Propionate**		
**Substrates**	**Acetate**	**Propionate**	**Mixture**	**Acetate**	**Propionate**	**Mixture**

*Y*_*X*, *Ac*/*S*_	0.66 (0.01)	-	0.72 (0.03)	0.52 (0.03)	-	0.50 (0.10)
*Y*_*X*, *Prop*/*S*_	-	0.56 (0.04)	0.78 (0.02)	-	0.51 (0.02)	0.57 (0.10)
*Y*_*PHB*/*S*_	0.76 (0.01)	0.75 (0.02)	0.84 (0.01)	0.68 (0.02)	0.71 (0.01)	0.71 (0.06)
*Y*_*PHV*/*S*_	-	0.73 (0.02)	0.80 (0.01)	-	0.70 (0.01)	0.69 (0.06)
*Y*_*PH*2*MV*/*S*_	-	0.72 (0.02)	0.78 (0.01)	-	-	-
*m*_*S*_	0.0037 (0.0002)	0.0091 (0.0010)	0.0034 (0.0003)	0.0065 (0.0008)	0.011 (0.001)	0.0079 (0.0025)
*Y*_*X*, *Ac*/*O*2_	1.90 (0.11)	-	2.00 (0.19)	1.09 (0.13)	-	0.86 (0.40)
*Y*_*X*, *Prop*/*O*2_	-	0.93 (0.12)	2.56 (0.26)	-	0.79 (0.04)	1.10 (0.51)
*Y*_*PHB*/*O*2_	5.06 (0.30)	2.28 (0.29)	5.83 (0.58)	2.92 (0.36)	1.94 (0.10)	2.51 (1.16)
*Y*_*PHV*/*O*2_	-	2.52 (0.32)	6.32 (0.63)	-	2.15 (0.11)	2.72 (1.26)
*Y*_*PH*2*MV*/*O*2_	-	2.71 (0.35)	6.69 (0.66)	-	-	-
*m*_*O*2_	0.0037 (0.0002)	0.011 (0.001)	0.0037 (0.0003)	0.0065 (0.0008)	0.0120 (0.0006)	0.0086 (0.0027)

The confidence bounds of the theoretical yields were obtained by error propagation of P/O ratio (δ)

### Metabolic flux distribution

**Figure 8:**
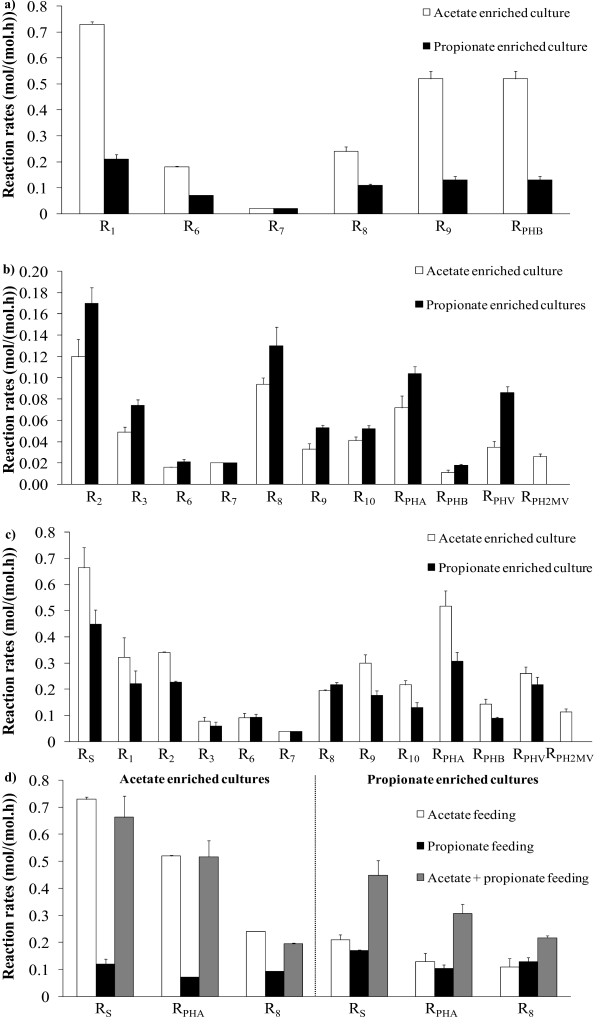
**MFD results**. Figure shows MFD results for acetate (a), propionate (b) and acetate + propionate (c) feeding using acetate (■) and propionate (□) enriched cultures. Figure 8d presents MFD results of the main substrates uptake, PHA and oxidative phosphorylation fluxes for the two enriched cultures.

The calculated metabolic flux distribution (MFD) was obtained in all cases in conditions of excess of carbon source material and negligible cell growth conditions (low ammonia concentration in the medium). The results are shown in Figures 8a–d. When acetate is fed as the sole carbon source to acetate or propionate enriched cultures, the overall metabolic activity is significantly higher in the former case than in the latter case (Figure 8a). All fluxes are at least two-threefold higher in the acetate enriched culture as compared to the propionate enriched culture.

On the other hand, if propionate is fed to both cultures, all fluxes, without exception, are higher in the propionate enriched culture when compared to the acetate culture (Figure 8b). These results confirm that the substrate used for culture enrichment is more effectively metabolized by the selected culture. However, the differences between the fluxes of both cultures in Figure (8b) are markedly lower than those of Figure (8a). In particular, the total carbon uptake and corresponding PHA storage are much higher in the acetate enriched culture fed with acetate. It seems that acetate was a much more effective substrate than propionate for selective enrichment of PHA producing cultures, leading to higher quantities of PHA stored.

The simultaneous feeding of acetate and propionate to the enriched cultures is shown in Figure 8c. Acetate and propionate are taken up at similar fluxes in each culture: 0.33 C-mol/(C-mol.h) of acetate and 0.34 C-mol/(C-mol.h) of propionate was taken up in acetate enriched cultures and 0.22 C-mol/(C-mol.h) of acetate and 0.23 C-mol/(C-mol.h) of propionate was taken up in propionate enriched cultures. The total carbon flux is 33% higher in acetate enriched cultures when compared to the propionate enriched cultures. The acetate enriched culture produced a total PHA flux of 0.52 C-mol/(C-mol.h), whereas the PHA flux in the propionate enriched culture is 0.31 C-mol/(C-mol.h) (40% lower). However, the catabolism flux, *R*_6_, is about the same in both cultures, suggesting that the percentage of carbon source spent for maintenance is higher for propionate enriched cultures due to the much lower P/O ratio in propionate enriched cultures. This is further confirmed by the higher oxidative phosphorylation flux, *R*_8_, for the propionate enriched culture.

It is also clear from Figures 8b–c that PH2MV was produced in the acetate culture and not in the propionate culture, due to the fact that acetyl-CoA* and propionyl-CoA* tended to condense randomly in the acetate culture and selectively in the propionate culture. A possible explanation for this result is that selective condensation of propionyl-CoA* with acetyl-CoA* may be a property of cells acclimatized to a propionate substrate, and that the acetate culture, which was never previously exposed to propionate, may have lacked the necessary enzymes or microbial population that is responsible for selective condensation.

Figure 8d shows a comparison of the fluxes for some of the main reactions in the two enriched cultures during the different feeding conditions. In acetate enriched cultures, the total VFA uptake capacity obtained with acetate feeding (0.73 C-mol/(C-mol.h)) was approximately equal to the sum of acetate and propionate uptake, when the substrates were fed simultaneously. Propionate enriched cultures showed a completely different behaviour. The total carbon uptake observed when both substrates were fed to this culture was approximately double that of the propionate or acetate uptake when the substrates were fed individually. A similar trend can be observed for the oxygen consumption flux and total PHA production flux, where the total flux in the propionate culture is approximately equal to the individual acetate and propionate fluxes combined, while the mixed substrate feed exhibited similar fluxes to the case of acetate feeding in the acetate culture. The mixed substrate feed had a synergistic effect on the individual metabolism of both substrates: both studied cultures were more able to take up propionate and convert it into PHA with a combined acetate/propionate feeding. This increase in VFA uptake was also observed in a culture adapted to a mixture of acetate, propionate and lactic acid [16]. A possible explanation for this result is that the conversion of propionyl-CoA to acetyl-CoA is the rate limiting step for PHV synthesis using only propionate. The effect of this limitation on VFA uptake rate is attenuated when acetate is also fed, because the requirement of propionyl-CoA driven to acetyl-CoA is lower and more propionyl-CoA can be driven for propionyl-CoA* synthesis without losing carbon in reaction, R_3_.

Since the fraction of propionate per total VFA in the feed had a substantial influence on the amount of propionate uptake driven to acetyl-CoA, a relationship was established based on these two fractions, for each mixed culture under either a propionate or a mixed propionate-acetate feed (see Eq. 12f). The linear regression coefficient, r^2^, for this equation was found to be 0.98, based on the data presented in Table 4. It is clear that an increase in the propionate fraction fed to the mixed cultures leads to an increase in the amount of propionate converted through propionyl-CoA to acetyl-CoA. Thus, there was a higher requirement for acetyl-CoA production when the relative acetate uptake rate is lower. This supports the hypothesis discussed above, where it was proposed that energy generation through the catabolic activity of the cells are preferentially executed through acetyl-CoA.

Overall, the results from the tests with the simultaneous feeding of acetate and propionate again corroborate the hypothesis that acetate was a much more effective substrate than propionate for the selection of an optimal PHA producing culture through enrichment via the feast and famine regimen.

### Flux balance analysis

From the results presented above, it is clear that acetate is a superior substrate to propionate for the enrichment of a microbial culture performing PHA production under the feast and famine regimen, due to the higher oxidative phosphorylation efficiency with this carbon source, resulting in higher PHA productivity. It is also clear that the feeding of both acetate and propionate carbon sources simultaneously resulted in either a similar or higher total PHA productivity, with a higher diversity of 3HA monomers as compared to the feeding of a single substrate. Thus, in PHA producing systems where the microbial culture enrichment and PHA production phases are separated, it is desirable to enrich the microbial culture using acetate, while feeding a combination of acetate and propionate in the PHA production phase.

**Figure 9:**
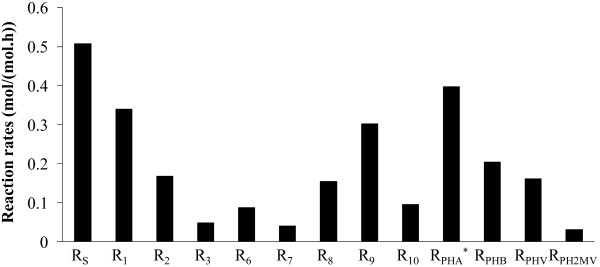
**FBA results**. Figure shows FBA results obtained by maximizing the PHA flux (RPHA*) with a propionyl-CoA* content of 24% (C-mol basis).

During the PHA production phase, it is possible to control the final PHA polymer composition through manipulating the acetate and propionate feeding fractions, using the metabolic model presented in this study. It was stated that a PHA copolymer with 15–20 mol % of propionyl-CoA* (18–24 C-mol %) is desired for improved toughness of the final polymer [18]. Assuming this propionyl-CoA* content as a constraint, the optimal feeding strategy required to obtain this copolymer was defined by performing FBA on the acetate enriched culture, where Eq. 12f was also used as a constraint. The FBA results are presented in Figure 9. These results show that the maximum PHA production rate with the desired propionyl-CoA* content is obtained when the propionate uptake rate is below the maximum calculated from parameter estimation (0.34 C-mol/(C-mol.h)). The limitation of the propionate uptake rate can be achieved by ensuring that the feeding rate of propionate is equal to the desired propionate uptake rate during reactor operation. When the process was operated at the maximum propionate uptake rate, the obtained propionyl-CoA* content in the PHA polymer was about 42%.

### Metabolic comparison of PHA production by pure and mixed cultures

The process efficiency for PHA production by mixed microbial cultures was evaluated through a comparison of the metabolic fluxes for the PHA production process by pure cultures of microorganisms using the same carbon substrates (i.e. acetate and propionate). In the present study using mixed microbial cultures, the biomass growth was assumed to be negligible based on the very low feed concentration of ammonia. In the pure culture studies [18,37], the maximum carbon fraction used for biomass growth was less than 15%, providing a good basis for comparison with the present study. MFA was performed for a pure culture of *Cupriavidus necator *using acetate as the sole carbon source [37]. As shown in Table 6, the acetate uptake rate is higher for mixed microbial cultures enriched on acetate (0.73 C-mol/(C-mol.h)) than for pure cultures (0.44 C-mol/(C-mol.h)). However, the acetate uptake rate in the cultures enriched on propionate is half of the rate observed in pure cultures (0.21 C-mol/(C-mol.h)). The fluxes for CO_2 _formation and O_2 _consumption are slightly higher for pure cultures. These differences could be explained by the additional energetic consumption for cell growth in pure cultures that is negligible in both mixed cultures. The fraction of carbon driven for PHB is significantly higher for both mixed cultures. This is likely due to the very high P/O ratio observed, especially in the mixed culture fed with acetate and, hence, a more efficient utilization of the carbon supply is expected.

**Table 6:** Fluxes distribution for acetate and acetate + propionate feeding between mixed microbial cultures and *Cupriavidus necator*

**Feeding**	**Acetate**			**Acetate + Propionate**		
**Culture enrichment**	**Acetate**	**Propionate**		**Acetate**	**Propionate**	
			
**Cultures**	**Mixed microbial cultures (This work)**		***Cupriavidus necator ***[37]	**Mixed microbial cultures (This work)**		***Cupriavidus necator ***[18]

	**Substrate uptake rate (C-mol/(C-mol.h))**					

**VFA uptake rate**	0.73	0.21	≈ 0.44^1^	0.67	0.45	≈ 0.38^1^
**Acetate uptake rate**				0.33	0.22	≈ 0.16^1^
**Propionate uptake rate**	-	-	-	0.34	0.23	≈ 0.22^1^

	**Carbon and oxygen flux distribution (%)**					

**Carbon converted to growth**	0.7	3.3	15.0	2.0	3.3	8.7
**Carbon converted to CO**_2_	25.7	35.0	37.8	20.0	28.1	50.1
**Oxygen consumed per carbon**	16.5	27.3	19.8	14.7	24.3	-
**Carbon converted to PHA**	73.6	61.7	47.2	78.0	68.6	41.2
Carbon converted to Ac-CoA*	73.6	61.7	47.2	45.2	39.5	36.9
Carbon converted to Propionyl-CoA*	-	-	-	32.8	29.1	4.3

^1^Approximate rates estimated from experimental results

For the case of a *Cupriavidus necator *culture fed with a mixture of acetate and propionate [18], the VFA uptake kinetics were observed to be different from the mixed microbial cultures enriched with either acetate or propionate, despite the fact that the initial fraction of acetate and propionate were the same in all cases (50:50 on a C-mol basis). The VFA uptake rate observed in the acetate enriched culture is much higher than in pure cultures (about twofold), whereas, the propionate enriched culture has only a slightly higher VFA uptake rate as compared to the pure cultures. The PHA production fluxes were observed to be much higher for the cases of the mixed cultures (78.0 and 68.6%), as compared to the pure culture (41.2%), during the feeding of a mixture of acetate and propionate. Additionally, despite the fact that the relative propionate uptake rate is higher in pure cultures (63%) than in mixed cultures (51%), the fraction of propionyl-CoA driven to propionyl-CoA* is significantly lower (86% lower). This is due to the fact that a large portion of the propionate fed to *Cupriavidus necator *was first converted to acetyl-CoA prior to PHA production, unlike the mixed culture cases. This metabolic pathway causes a large loss of CO_2 _by the pure culture, thus lowering the PHA production efficiency. Furthermore, a large fraction of this propionyl-CoA converted to acetyl-CoA was then used for cell catabolism, contributing to the much higher carbon flux observed in the case of the pure culture as compared to the mixed cultures.

It should be noted that the carbon source used in pure culture studies [18,37] was neither acetate nor propionate, but mixtures of yeast extract, meat extract and peptone. This could be one explanation for the inferior VFA uptake and PHA production results observed in these studies as compared to the present study, since the culture may require an adaptation period to synthesize the necessary enzymes for the metabolism of acetate and propionate carbon sources. Alternatively, it may be that the microorganisms present in the mixed cultures are more efficient PHA producers when compared to the pure cultures of *Cupriavidus necator*, due to the feast and famine regimen imposed. An increased VFA uptake rate was observed for a pure culture of *Amaricoccus kaplicensis *when the length of the famine period was increased [38].

## Conclusion

This paper presents a metabolic model of PHA copolymers production in mixed microbial cultures. The model was applied to two different cultures, obtained through two distinct enrichment protocols (selected with either acetate or propionate), under different feeding conditions (fed with either a single substrate or with mixtures of the two substrates). With this model, intracellular flux distributions for the different cases were calculated. Also, feeding scenarios were optimized by FBA targeting maximal productivity of a copolymer with a desired monomeric composition. These results were benchmarked with published results of pure cultures of *Cupriavidus necator*. From these studies the following main conclusions may be highlighted:

• The substrate used for culture enrichment by feast and famine feeding has a high selective pressure on the organisms that compose the final culture in the sense that the organisms selected are those that most effectively metabolize the adopted substrate.

• Sludge enrichment by acetate feeding through feast and famine regimen is much more selective towards a culture with high PHA storage fluxes and yields than when propionate is used.

• The P/O ratio is highly dependent on the substrate and the microbial culture selected. Acetate metabolism has a consistently higher P/O ratio (close to the theoretical maximum of 3 mol-ATP/mol-NADH_2_) than propionate metabolism.

• MFA studies suggest that when mixtures of acetate and propionate are fed to the cultures, the catabolic activity is primarily guaranteed through acetate uptake. Coherently, the acetate P/O ratio tends to prevail over that of propionate when both substrates are fed simultaneously.

• The application of FBA targeting the optimization of the PHA synthesis flux with a 24% (C-mol/C-mol) propionyl-CoA* composition revealed that acetate should be fed in excess whereas the feeding of propionate should be limited to ~0.17 C-mol/(C-mol.h). Under excess of both substrates the final propionyl-CoA* content is around 42% (C-mol/C-mol).

• By comparing metabolic fluxes between mixed and pure cultures of *Cupriavidus necator *it can be concluded that the PHA production process by mixed microbial cultures has the potential to be comparable or even more favourable than what is achieved by pure cultures.

Although the metabolic characterization of mixed cultures was shown in this study to be highly favourable and promising, it should be noted that these results were obtained under ammonia limitation, thus, with conditions where cells growth is negligible. The compatibility of high cell growth rates with high PHA synthesis fluxes in mixed cultures is still not undoubtedly demonstrated in the literature.

## Abbreviations

*Acronyms*: **ASM3**: activated sludge model No. 3; **DO**: dissolved oxygen; **FBA**: flux balance analysis; **GAO**: glycogen-accumulating organisms; **3HA**: 3-hydroxyalkanoate; **3HB**: 3-hydroxybutyrate; **3H2MB**: 3-hydroxy-2-methylbutyrate; **3H2MV**: 3-hydroxy-2-methylvalerate; **3HV**: 3-hydroxyvalerate; **HRT**: hydraulic retention time; **MFA**: metabolic flux analysis; **MFD**: metabolic flux distribution; **PAO**: polyphosphate-accumulating organisms; **PHA**: poly-hydroxyalkanoates; **PHB**: poly(3-hydroxybutyrate); **PHV**: poly(3-hydroxyvalerate); **PH2MV**: poly(3-hydroxy-2-methylvalerate); **P/O ratio**: ATP synthesis/oxygen consumption ratio; ***rmse***: root mean squared error; **SBR**: sequencing batch reactor; **SRT**: sludge retention time; **TCA**: tricarboxylic acid cycle; **VFA**: volatile fatty acids; **VSS**: volatile suspended solids. *Symbols*: **Ac**: acetate/acetate concentration (C-mmol/l); **f**_**i**_: intracellular PHA, PHB, PHV and PH2MV contents (C-mol/C-mol); **f**_**PHA, max**_: maximum intracellular PHA contents (C-mol/C-mol); **K**_1_, **K**_2_: energy requirements for biomass synthesis (mol-ATP/C-mol); **K**_**S**_: acetate and propionate half-saturation constants (C-mmol/l); **K**_**N**_: ammonia half-saturation constant (N-mmol/l); **m**_**j**_: maintenance on component j [C-mol/(C-mol.h)]; **m**_**ATP**_: maintenance coefficient on ATP [mol-ATP/(C-mol.h)]; **N**: ammonia/ammonia concentration (N-mmol/l); **O**_2_: oxygen/oxygen concentration (mmol/l); **Prop**: propionate/propionate concentration (C-mmol/l); ***R*_*i*_**: specific rate of reaction on compound i [C-mol/(C-mol.h)]; ***R*_*i*, *max*_**: maximum specific rate of reaction on compound i [C-mol/(C-mol.h)]; **S**: total VFA/total VFA concentration (C-mmol/l); **t**: culture runtime (h); **X**: active biomass/active biomass concentration (C-mmol/l); **y**: fraction of propionate uptake rate in the total VFA uptake rate; **Y_i/j_**: yield of component i on component j (C-mol/C-mol); **α**: PHB production saturation order constant (dimensionless); **δ**: efficiency of oxidative phosphorylation (mol-ATP/mol-NADH_2_).

## Authors' contributions

All authors read and approved the final manuscript. JMLD and RO developed the software. JMLD, AO and RO participated in the model implementation. LSS and PCL performed experimental work. MAMR and RO designed and coordinated the study. JMLD, AO and RO drafted the manuscript.
